# Erysipelothrix spp. and other Erysipelotrichales detected by 16S rRNA microbial community profiling in samples from healthy conventionally reared chickens and their environment

**DOI:** 10.1099/acmi.0.000736.v3

**Published:** 2024-06-05

**Authors:** Eva Wattrang, Tina Sørensen Dalgaard, Helena Eriksson, Robert Söderlund

**Affiliations:** 1 Department of Microbiology, Swedish Veterinary Agency, Uppsala, Sweden; 2 Department of Animal and Veterinary Sciences, Aarhus University, Tjele, Denmark; 3 Department of Animal Health and Antimicrobial Strategies, Swedish Veterinary Agency, Uppsala, Sweden

**Keywords:** erysipelotrichales, *Erysipelothrix* spp., chicken

## Abstract

Outbreaks of erysipelas, a disease caused by infection with *Erysipelothrix rhusiopathiae* (ER), is a re-emerging problem in cage-free laying hen flocks. The source of ER infection in hens is usually unknown and serological evidence has also indicated the presence of ER or other antigenically related bacteria in healthy flocks. The aim of the present study was to evaluate sample collection, culture methods and DNA-based methodology to detect ER and other Erysipelotrichales in samples from healthy chickens and their environment. We used samples from a research facility with conventionally reared chickens with no history of erysipelas outbreaks where hens with high titres of IgY recognising ER previously have been observed. Microbial DNA was extracted from samples either directly or after pre-culture in nonselective or ER-selective medium. Real-time PCR was used for detection of *Erysipelothrix* spp. and high-throughput amplicon sequencing of 16S rRNA sequencing was used for detection of Erysipelotrichales. A pilot serological analysis of some Erysipelotrichales members with IgY from unvaccinated and ER-vaccinated high-biosecurity chickens, as well as conventionally reared chickens, was also performed. All samples were negative for ER, *E. tonsillarum* and *E. piscisicarius* by PCR analysis. However, 16S rRNA community profiling indicated the presence of several Erysipelotrichales genera in both environmental samples and chicken intestinal samples, including *Erysipelothrix* spp. that were detected in environmental samples. Sequences from *Erysipelothrix* spp. were most frequently detected in samples pre-cultured in ER-selective medium. At species level the presence of *Erysipelothrix anatis* and/or *Erysipelothrix aquatica* was indicated. Serological results indicated that IgY raised to ER showed some cross-reactivity with *E. anatis*. Hence, environmental samples pre-cultured in selective medium and analysis by 16S rRNA sequencing proved a useful method for detection of Erysipelotrichales, including *Erysipelothrix* spp., in chicken flocks. The observation of such bacteria in environmental samples offers a possible explanation for the observation of high antibody titres to ER in flocks without a history of clinical erysipelas.

## Data Summary

All results from the presented experiments are included in the published article, and sequence data with corresponding sample metadata are available from the European Nucleotide Archive https://www.ebi.ac.uk/ena(https://www.ebi.ac.uk/ena) under project number PRJEB67586.

The authors confirm all supporting data and protocols have been provided within the article or through supplementary data files.

## Introduction

The family *Erysipelotrichaceae* in the order Erysipelotrichales currently comprises several genera, with *Erysipelothrix* considered the family type genus [1]. *Erysipelothrix rhusiopathiae* (ER), a Gram-positive facultative anaerobic rod, is probably the best-known bacterium of the family. This bacterium was first described in the late nineteenth century and is known to infect a large variety of species, including mammals, birds and fish, with or without causing clinical disease [2]. Within the genus *Erysipelothrix, E. tonsillarum* was described in 1987 [3] and is considered non-pathogenic in pigs and chickens [3,5] but a potential pathogen in dogs [6]. Among more recently described species in this genus, *E. piscisicarius* has been associated with disease in pigs, turkeys and ornamental fish [7,8], while *E. inopinata* [9], *E. larvae* [10], *E. anatis*, *E. aquatica* and *E. urinaevulpis* [11] have not yet been reported to be associated with disease.

The disease caused by ER in animals is termed erysipelas and among livestock this disease is most commonly recognised as a problem in pig and turkey production [2]. However, erysipelas is also considered an emerging disease in modern egg production [12,20]. The emergence of the disease in laying hens has been associated with the move from cage to floor housing and housing systems with outdoor access for hens have an increased risk of erysipelas outbreaks [14,16]. Outbreaks of erysipelas in laying hen flocks are characterised by acute onset of disease with rapid progression and up to 60 % mortality and may include decreased egg production [13,16, 17, 21]. The source and route of infection of outbreaks are most often not known. The progression of erysipelas outbreaks in laying hen flocks would suggest the introduction of a pathogen in a naïve population. However, several reports have shown antibodies that recognise ER in conventionally reared laying hens with no history of erysipelas [15,22,24], which suggests that ER or antigenically similar bacteria could be common in these chickens or in their environment. However, there are only a few reports of isolation of ER from clinically healthy chickens [25,27] and additionally there is a study that failed to isolate ER by culture methods in healthy laying hen flocks [18]. By whole-genome sequencing we have found (manuscript in preparation) that ER from laying hens affected by clinical erysipelas belong to all described [28] genetic clades except clade 1. Moreover, we have observed that clade 1 was the most common ER in samples from healthy pigs and wild boar [29]. This ER clade has previously been associated with disease in marine mammals [28] and it is possible that ER of clade 1 is less pathogenic for terrestrial mammals and chickens. One may hypothesise that less pathogenic genotypes of ER may be the reason that ER-recognising antibodies are identified in healthy laying hens. It is known that under favourable conditions ER may survive in the environment for weeks [30], but an important role of carriers as reservoirs and source of ER for clinical outbreaks of erysipelas has also been put forward [31]. To better understand the pathogenesis of erysipelas in laying hens as well as to be able to prevent this disease, it is important to have a means to monitor the presence of ER and other Erysipelotrichales in healthy hens and their environment. This has proven to be challenging by traditional culture methods because low-level presence of ER is easily missed in samples where faster-growing bacterial species are prevalent. Hence, DNA-based methodologies might prove beneficial for this purpose.

To gain more insight into the presence of ER and related bacteria in healthy chickens and their environment, this study was conducted to evaluate sample collection, culture methods and DNA-based methodology to detect Erysipelotrichales. For this purpose, samples were collected from an experimental poultry facility with a conventional biosecurity level. The facility had no history of erysipelas outbreaks but healthy chickens with high IgY titres recognising ER had previously been identified (unpublished observation). In addition, a pilot serological analysis was performed using some Erysipelotrichales members with IgY from specific pathogen-free (SPF) and conventionally reared chickens to monitor the presence of IgY recognising these bacteria.

## Methods

### Sample collection for bacterial DNA analysis

Samples were collected at the experimental poultry facility at Foulum research centre, Aarhus University (AU), Denmark (Fig. S1, available in the online version of this article). At this facility research chicken lines are bred and maintained, with chicks hatched from eggs produced in-house and raised to adulthood. Adult chickens are group housed, seven hens and one rooster, in furnished cages. Other poultry are kept at the facility for temporary experiments, and other livestock, including pigs, are kept in nearby buildings at the same research centre. The facility biosecurity is rated as ‘conventional production’, i.e. not SPF. No outbreaks of erysipelas have occurred at the facility, but high antibody titres to ER among adult hens have been observed (authors’ unpublished observations). A total of 56 samples were collected (Table 1). Four 10–12-week-old chickens were sacrificed, and intestinal segments with content were collected from the ileum, caecum and cloaca (total *n*=12). Environmental samples were collected with gauze wipes for surfaces and gauze overshoes for floor and ground samples; in both cases the gauze was moistened with phosphate-buffered saline (PBS) before use. Samples were collected from cages (*n*=12), manure conveyor belts (*n*=12), floors in the animal rooms (*n*=8), facility staff shoes used in the animal room (*n*=2), the ground outside the poultry facility (*n*=4) and the ground outside the pig house (*n*=4). Pooled samples of poultry red mites (*Dermanyssus gallinae*), a suspected ER vector [12], were also collected (*n*=2).

**Table 1.** Genus-level detection of Erysipelotrichales in 56 samples from 10–12-week-old chickens and their environment as determined by 16S rRNA community profiling. Percentage positive samples includes all three sample treatment protocols

	Chicken samples (% pos.)			Environmental samples (% pos.)						
Genus	Ileum *n*=4	Caecum *n*=4	Cloaca *n*=4	Cages *n*=12	Manure belts *n*=12	Mites *n*=2	Outside pig stable *n*=4	Shoes *n*=2	Floors *n*=8	Outside poultry facility *n*=4
*Turicibacter*	100	0	75	92	33	0	50	100	75	25
*Holdemania*	0	0	0	8	17	0	0	0	0	0
*Erysipelothrix*	0	0	0	33	83	0	0	50	0	0
*Massiliomicrobiota*	100	75	100	8	42	0	0	0	0	0
*Erysipelatoclostridium*	50	100	100	100	75	100	50	50	50	25
*Longicatena*	0	0	0	8	17	0	0	0	0	0
*Merdibacter*	0	0	75	0	17	0	0	0	0	0
*Faecalitalea*	0	50	75	25	25	0	0	0	0	0
*Dielma*	0	100	75	25	42	0	0	0	0	0
*‘Clostridium’innocuum* group	0	0	0	8	8	0	0	0	0	0

### Enrichment and DNA extraction

All samples were divided into three portions used for the following protocols: (1) no enrichment, suspension in PBS; (2) nonselective enrichment, 48 h incubation at 37°C in tryptic soy broth (TSB); and (3) selective enrichment, 48 h incubation at 37°C in beef extract broth containing 0.2 mg ml^−1^ sodium azide and 5 µg ml^−1^ crystal violet (SACV). Growth media were prepared at the Swedish Veterinary Agency (SVA; Uppsala, Sweden). In all three protocols, the amount of liquid added was approximately 10× the volume of the sample. Before collection of supernatants the PBS suspensions, as well as the liquid cultures, were vortexed and large particles were allowed to sediment. Supernatants from the three protocols, a total of 168 samples, were frozen at −70°C. Bacteria were lysed in thawed supernatants by bead-beating the liquid with 0.1 mm zirconia/silica beads (BioSpec Products) for 2 min at 6.5 m s^−1^ in a FastPrep24 (MPBiomedicals). DNA was subsequently extracted from 200 µl aliquots using the IndiMag Pathogen kit (Indical) on a Maelstrom-9600 automated system (TANBead).

### Real-time PCR detection of *Erysipelothrix* spp.

A previously described fluorescent probe-based real-time PCR method [32] was used to detect ER, *E. tonsillarum* and *E. piscisicarius* (previously designated *E*. sp. ‘strain 2’ [8]). ER and *E. tonsillarum* positive controls were used in the assay, however no positive control for *E. piscisicarius* was available for the present study. The assay (Fig. S2) was run using PerfeCTa Multiplex qPCR ToughMix reagents (Quantabio) on a CFX96 Touch Real-Time PCR instrument (Bio-Rad). To deliberately reduce the assay specificity in order to detect any other closely related *Erysipelothrix* spp. in the samples a second PCR reaction was performed for each sample excluding the probes and instead running the assay with PerfeCTa SYBR Green SuperMix (Quantabio) and evaluating any PCR products by melting curve analysis (Fig. S2) and Sanger sequencing.

### 16S rRNA sequencing detection of Erysipelotrichales

Partial 16S sequencing for community profiling was performed using the Illumina MiSeq 16S rRNA protocol [33], with libraries verified on a 2100 Bioanalyzer instrument with a High Sensitivity DNA kit (Agilent) and sequenced as 2×300 bp paired-end reads on a with a V3 run kit on a MiSeq instrument (Illumina). The produced sequences from chicken and environmental samples were analysed separately using the QIIME2 16S pipeline [34] implemented on the NIH/NIAID Nephele microbiome analysis platform v2.20.2 [35] using the silva (RRID:SCR_006423) reference database with open OTU strategy and otherwise default settings. The output was processed and visualised with the phyloseq package v1.38.0 [36] in R 4.0.4 (r-project.org). To avoid spurious detections due to index jumping [37], all OTU counts below five in a sample were considered negative for that sample. A phylogenetic tree was inferred in mega X v10.0.1 [38] using the neighbour-joining method with 1000 bootstrap replicates, number of differences as distance metric and pairwise deletion of missing positions, comparing all sequences from putative Erysipelotrichales detected in the present study with selected sequences from the reference database, all trimmed to the length of the Illumina protocol PCR product. To find any matches in less well-curated sources all sequences from putative *Erysipelothrix* spp. as identified by QIIME2 were also compared to the full NCBI nr nucleotide collection with blastn (blast.ncbi.nlm.nih.gov). Genomes of interest identified in the 16S analysis were screened for the presence of genes encoding ER proteins with known or suspected immunogenicity using local blast+ running the blastn algorithm with default settings [39].

### Samples for serological analyses

Serum samples from chickens reared at either of two types of biosecurity levels were included in the study; SPF-reared chickens and chickens conventionally reared at the AU facilities described above. The SPF chickens were female Dekalb White Leghorn-type layer hybrids purchased from a commercial hatchery and raised from 1 day old at high biosecurity at SVA (Uppsala, Sweden) as earlier described [40]. In the current study we used sera collected from six unvaccinated chickens, two 45 days old (chickens #2 and #3) and four 52 days old (chickens #21, #26, #33 and #68), and six vaccinated chickens, two 45 days old (chickens #29 and #32) and four 52 days old (chickens #7, #16, #34 and #60). The vaccinated chickens were injected with 0.5 ml of a commercial inactivated erysipelas vaccine containing the ER strain M2 of serotype 2, belonging to clade 2, (Porcilis ERY Vet, MSD Animal Health) in the breast muscle 28 days prior to sample collection (chickens #29 and #32) or 25 days prior to sample collection (chickens #7, #16, #34 and #60).

Conventionally reared chickens were mixed sexes inbred L10 [41,42] layer hybrid type hatched and reared indoors without outdoor access at the AU animal facilities. After hatching, the chickens were kept in floor pens on wood shavings until 16 weeks of age and subsequently transferred to enriched cages. In the current study, sera collected at 10 weeks of age (male, *n*=4) and at 48 weeks of age (1 female and 3 male, *n*=4) were used.

### ELISA methodology for quantification of antibodies in chicken serum

A previously described in-house ELISA for quantification of IgY titres to ER in chicken serum samples was used [40]. In brief, the target-coating antigen used was a preparation of whole bacteria that was sonicated and the lysate subsequently cleared of particulate matter by centrifugation according to a previously described protocol [40]. Each serum sample tested was titrated in at least seven twofold dilutions starting at dilutions between 1 : 50 to 1 : 2000 depending on antibody concentration in the sample to achieve a dilution curve with a linear portion. This was performed for each of the three antigens used. For each sample the A450–A650 values were plotted against the sample dilution and the equation for the linear part of the curve was determined by regression analysis. Antibody titres were then calculated as the dilution that would achieve an A450–A650 value of 1 (Fig. S3). In the current study either antigens prepared from ER, *Holdemania filiformis* or *E. anatis*, respectively, were used as coating antigens. The ER antigen was prepared from strain 15-ALD003475 derived from an outbreak of erysipelas in a Swedish laying hen flock in 2015 that was classified as belonging to ‘intermediate’ lineage [28] and according to *in silico* application of multiplex PCR serotyping [43] it is of serotype 1B. A reference strain of *H. filiformis* (CCUG 39501T) was purchased from the Culture Collection, University of Gothenburg and cultured anaerobically according to a protocol from the supplier. A reference strain of *E. anatis* (DZM 111258) was purchased from Leibniz‐Institut DSMZ and cultured at microaerophilic conditions according to a protocol from DSMZ. The *H. filiformis* and *E. anatis* antigens were subsequently prepared according to the same protocol described for the ER antigen [40]. The protein concentrations of all antigens were determined by Bradford protein assay and for coating a final concentration of 5 μg ml^−1^ was used for all antigens.

## Results

All chicken intestinal samples as well as all environmental samples were negative for ER, *E. tonsillarum* and *E. piscisicarius* by real-time PCR, and no positive reactions in the SYBR Green assay indicated the presence of closely related species. As expected from the heterogenous sample material, 16S rRNA sequencing produced variable read counts from the different categories. The chicken intestinal tract sampling resulted in a total of 38405–171 517 high-quality sequences after filtering from each animal combining the three pre-treatment protocols. The two poultry red mite samples yielded low sequence counts of 7807 and 8923. In contrast, more sequence data were retrieved from all environmental sample categories with read counts ranging from 109 283 to 414 161 per sample. A single sample from a manure conveyor belt (#89, TSB treatment) failed to produce any sequence data. As expected, either of the two enrichment protocols improved overall sequence recovery across all sample categories with the exception of the poultry red mite samples. QIIME2 analysis revealed the presence of several Erysipelotrichales genera in both environmental samples and chicken intestinal samples, including *Massiliomicrobiota*, *Erysipelatoclostridium*, *Turicibacter*, *Merdibacter*, *Faecalitalea* and *Dielma*. The environmental samples additionally contained representatives of *Longicatena*, the ‘*Clostridium*’ *innocuum* group, *Holdemania* and *Erysipelothrix* (Table 1, Fig. S4). The sequences consistent with the genus *Erysipelothrix* (Fig. S5) were found in a single shoe sample, 4 chicken cage samples and 10 samples from manure conveyor belts. The selective SACV enrichment was the most successful in producing *Erysipelothrix*-positive samples (14 samples), followed by PBS (7 samples) and TSB (1 sample). The fraction of observed sequences consistent with the genus *Erysipelothrix* was ≤1 % of the total sequence count for all these samples.

Consistent with real-time PCR results, no 16S sequences indicated the presence of the better-known *Erysipelothrix* species (Fig. S4). However, blast searches against the nr sequence database revealed close matches. *Erysipelothrix* sequence #2 in the present study is highly similar (99.6 %) to the 16S sequences of the two recently proposed novel species, *E. anatis* sp. nov. and *E. aquatica* sp. nov [11]. These species are closely related and cannot be differentiated by their 16S sequences [11]. Matches in GenBank to this genotype included isolates from pigs, geese, ducks and a turtle, as well as two species of beetle and a leech. No other putative *Erysipelothrix* sequences found in the present study matched any bacterial isolate in the database. However, sequence #1 closely (99 %) matches uncultured bacteria in swine waste (GenBank GQ138120.1), and sequences #3 and #7 have a high similarity (99 %) to uncultured bacteria in a leafhopper (KC864783.1) and a manure sample (KP745023.1). For the remaining *Erysipelothrix* sequences, no relevant matches (>97 %) were found.

The genomes of *E. anatis* sp. nov. and *E. aquatica* sp. nov. were investigated for the presence of sequences similar to those encoding putative immunogenic proteins in ER. For both genomes, GAPDH was present and similar to ER (100 % coverage, 87 % similarity in both). Genes similar to ER *Atsp* (92–98 % coverage, 73–74 % similarity) and to a lesser degree *dap2* (51–53 %, 69–71 %) were also found in both genomes, whereas no close matches to the genes *spaA*, *rspA*, *CbpB*, *Plp*, *Neu*, *Bga*, *Bml*, *CwpA*, or *CwpB* were found.

**Fig. 1.**
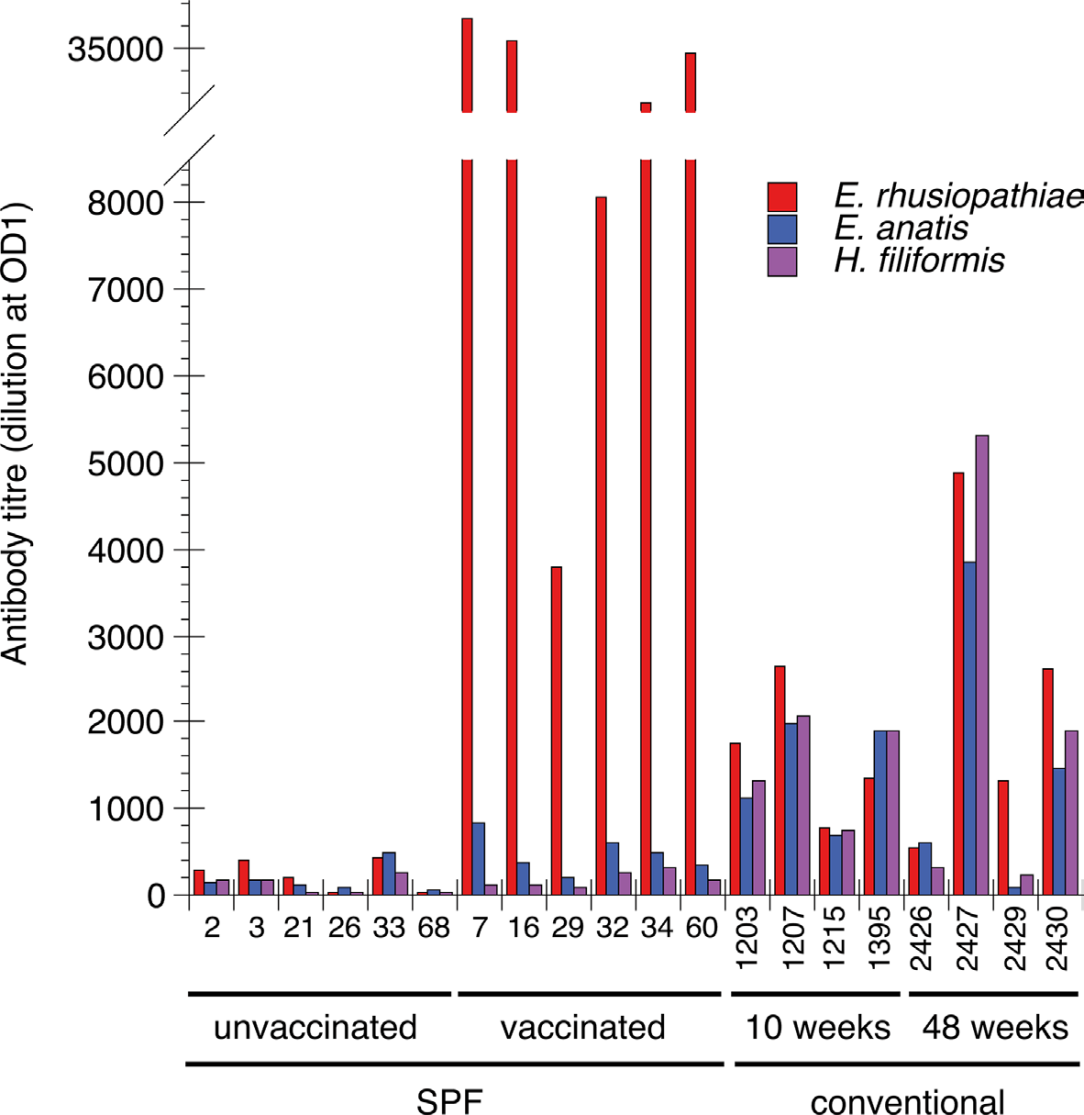
Quantification of IgY to ER (red bars), *E. anatis* (blue bars) and *H. filiformis* (purple bars) using ELISA methodology in sera from SPF-raised chickens (6–7 weeks old), unvaccinated and vaccinated against ER, respectively, and conventionally reared chickens at the AU research facilities (for details see Methods). Data are individual values for the indicated chickens.

Serological analysis of IgY antibodies to ER, *E. anatis* and *H. filiformis* was performed using ELISA methodology on samples from SPF-reared chickens as well as from chickens reared at the AU research facilities (Fig. 1). For unvaccinated SPF chickens reared under high biosecurity low levels of IgY that recognised either bacterium were detected. However, the SPF chickens that had received a dose of a commercial vaccine against erysipelas showed high IgY titres to ER, >10-fold higher than those of the unvaccinated SPF chickens. For the vaccinated SPF chickens a trend of slightly higher titres to *E. anatis* compared to those of unvaccinated SPF chickens was observed, while titres to *H. filiformis* were low. For the conventionally reared chickens from the AU research facilities variation between individuals in the levels of IgY titres to all three bacteria was observed. In general, the conventionally reared chickens showed higher titres compared to the unvaccinated SPF chickens and some individuals showed titres at comparable levels to those observed to ER for some of the vaccinated SPF chickens. However, unlike the vaccinated SPF chickens, for all conventionally reared chickens except #2429 the titre levels to all three bacteria within chicken were similar. Chicken #2429, on the other hand, showed a clear bias, with the highest titre to ER and low titres to the other two bacteria.

## Discussion

We have previously noted that chickens housed at the AU research facility have high serum titres of IgY that recognise ER, even though the facility has never experienced any outbreak of clinical erysipelas in chickens (unpublished observation). Given our recent observations of the occurrence of ER clade 1 in pigs and wild boar without clinical signs of disease [29] and the increasing number of novel related species discovered in a broad range of animal species in recent years [7,9,11], our hypothesis was that the presence of low-virulence *Erysipelothrix* sp. strains could explain the seropositive status of the chickens. Identifying such strains would be of interest both for better understanding ER evolution and pathogenesis as well as for improving management practices, e.g. vaccination programmes. Additionally, the authors have conducted several experimental ER infection studies in recent years and found that even when using a strain recovered from a severe outbreak in a production flock, infection under experimental conditions can be associated with limited or no clinical signs [40,44]. As an alternative hypothesis, virulent ER could thus be present at AU but not cause overt disease in the chickens due to host protective factors, e.g. a robust health status and low levels of stress. To determine if ER shed only intermittently or by a small number of animals we therefore performed extensive environmental sampling of the facility but could not detect ER DNA in any of the samples with real-time PCR or with 16S rRNA sequencing. The presence of ER among the animals therefore seems a more unlikely explanation, although the bacteria could of course have been present at an earlier date and cleared from the environment when sampled. It is also possible that ER was carried by a very small proportion of asymptomatic chickens at the time of sampling and that the number of chickens sampled thus was too small or that they were sampled at the wrong site. For example, *E. anatis* was isolated from the *sinus nasalis* of a duck and a goose, from a pig nose swab and from the brain of a goose [11], sites which were not included in the current sampling of chickens.

To detect *Erysipelothrix* spp. other than ER we used amplicon-based high-throughput 16S rRNA sequencing, amplifying a 460 bp fragment of the V3–V4 region with primers targeting conserved regions [33]. While unbiased, this approach produces only partial 16S sequences and can therefore not reliably identify bacteria to species level; previous studies have also found that even complete 16S sequences do not always provide good resolution between closely related *Erysipelothrix* species [8]. Nonetheless, we noted that multiple species of *Erysipelothrix* appeared to be present in environmental samples from the facility and in particular in chicken cages and on the chicken manure conveyor belts. A near-perfect match was, for instance, observed to *E. anatis*, which has been observed in ducks [11], and there were several sequences matching other undescribed and non-cultured bacteria related to the known species of *Erysipelothrix*. These observations are consistent with previous infection of some or all of the chickens with multiple species of *Erysipelothrix* without overt clinical signs, possibly due to lower virulence of these species or to protective host factors, e.g. a good general health status. Another possibility is the introduction of *Erysipelothrix* spp. directly to the cages via bedding material, feed, or arthropods. The highest number of samples positive for *Erysipelothrix* spp. was achieved with SACV broth, which is a long-standing standard protocol for enrichment of ER [45]. A tryptic soy variant of SACV broth has also been found to be the most efficient pre-enrichment for detection of ER by PCR in seafood samples [46]. This suggests that SACV can be useful in future efforts to isolate and further characterize some of the putative species of *Erysipelothrix* observed by us and in other studies. In the current study, however, even when samples were incubated in SACV the *Erysipelothrix* spp. read counts were low, representing a minute fraction of the bacteria in the enrichment broth. Thus, isolation of such low numbers of bacteria from highly contaminated samples by culture methods would likely have been challenging.

To assess potential antibody cross-reactivity of chicken IgY between ER and other *Erysipelothrix* species, consistent with the observations made in the 16S rRNA analysis, we performed quantification of IgY to ER, *E. anatis* and *H. filiformis* in sera from chickens raised at the AU facility and from SPF chickens raised under high biosecurity. *E. anatis* was chosen as a representative of *Erysipelothrix* species that were indicated at the AU facility. *H. filiformis* is the type species of *Holdemania* that were also present at the AU facility. *Holdemania* is one of the genetically closest genera to *Erysipelothrix* [1] and contains the uncommon type B cell wall murein in common with *Erysipelothrix* [47]. The results showed that chickens raised under high biosecurity had low titres to these three bacteria and under such circumstances vaccination with inactivated ER induced a clear IgY response with a very strong bias towards ER. IgY from ER-vaccinated SPF chickens showed a low but noticeable tendency to cross-react with *E. anatis*, which is in accordance with the bioinformatic analysis that showed that * E. anatis* shared some immunogenic proteins with ER but lacked, for instance, *spaA*, which is considered to be a major immunogen in ER [48]. The results from the conventionally reared chickens were, however, more difficult to interpret. With one exception, these chickens showed titres of similar levels to all three bacteria. Several explanations to this observation are possible, e.g. that infection/colonisation with live bacteria might induce more cross-reactive antibodies compared to vaccination with inactivated bacteria. It is also possible that natural, more polyreactive, antibodies [49] may be more common in the conventionally reared chickens. Further research is thus needed to understand the origin of the sometimes high titres of IgY recognising ER in conventionally reared chickens.

Taken together, the current results showed that environmental samples pre-cultured in selective medium and analysis by 16S rRNA sequencing proved a useful method for detection of Erysipelotrichales, including *Erysipelothrix* spp., in chicken flocks.

## supplementary material

10.1099/acmi.0.000736.v3 Supplementary Material 1.

## Abbreviations

ER: *Erysipelothrix rhusiopathiae*
PBS: phosphate-buffered saline
SACV: sodium azide crystal violet
SPF: special pathogen free
TSB: tryptic soy broth
