# Supplementary material for: Erysipelothrix spp. and other Erysipelotrichales detected by 16S rRNA microbial community profiling in samples from healthy conventionally reared chickens and their environment
Source: Access Microbiol. 2024 Jun 5;6(6):000736.v3. doi: 10.1099/acmi.0.000736.v3 (PMC11261693; doi:10.1099/acmi.0.000736.v3)
Supplement: Supplementary Material 1. [file acmi-6-00736-s001.pdf]

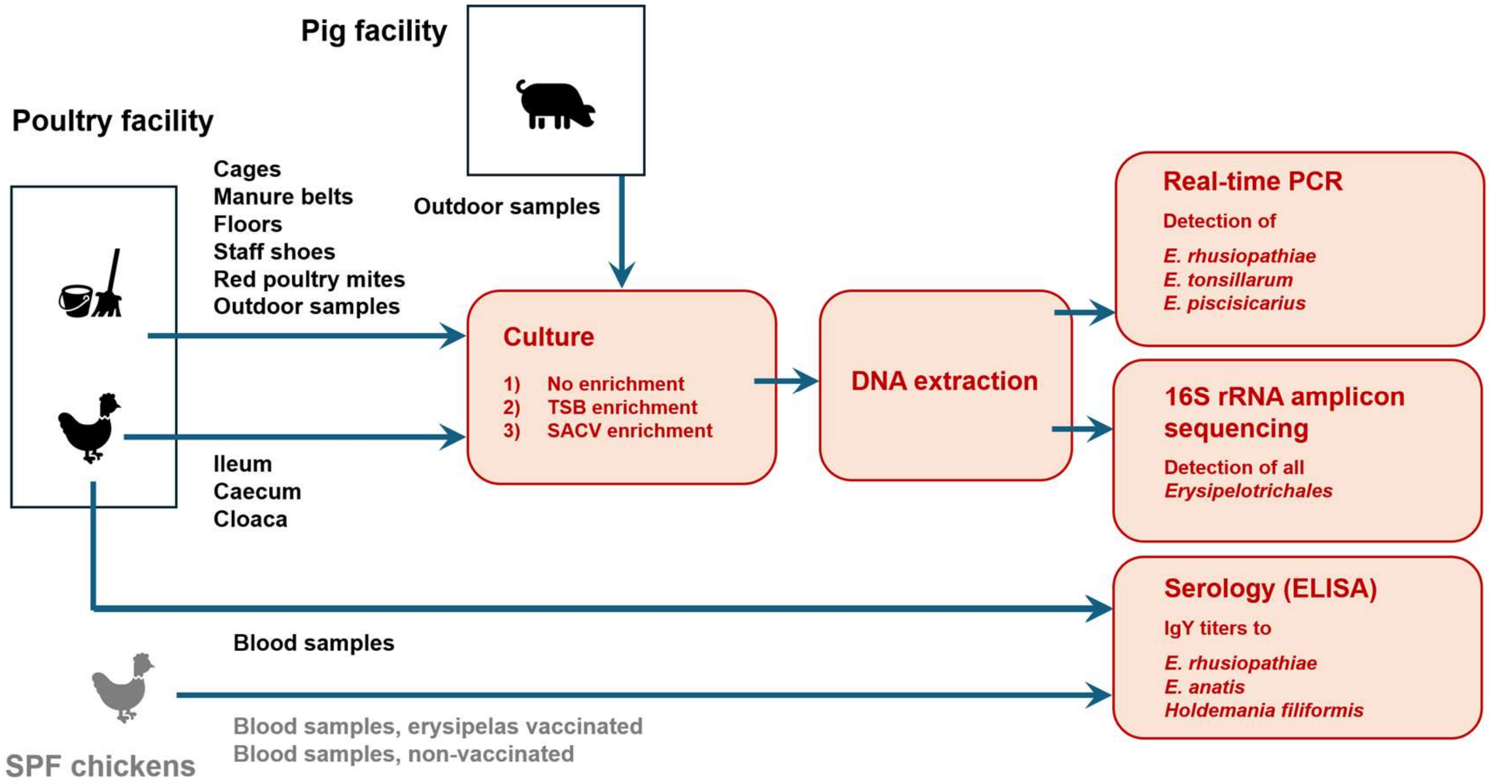

Supplementary figure 1. Outline of samples collected and analysis methods.

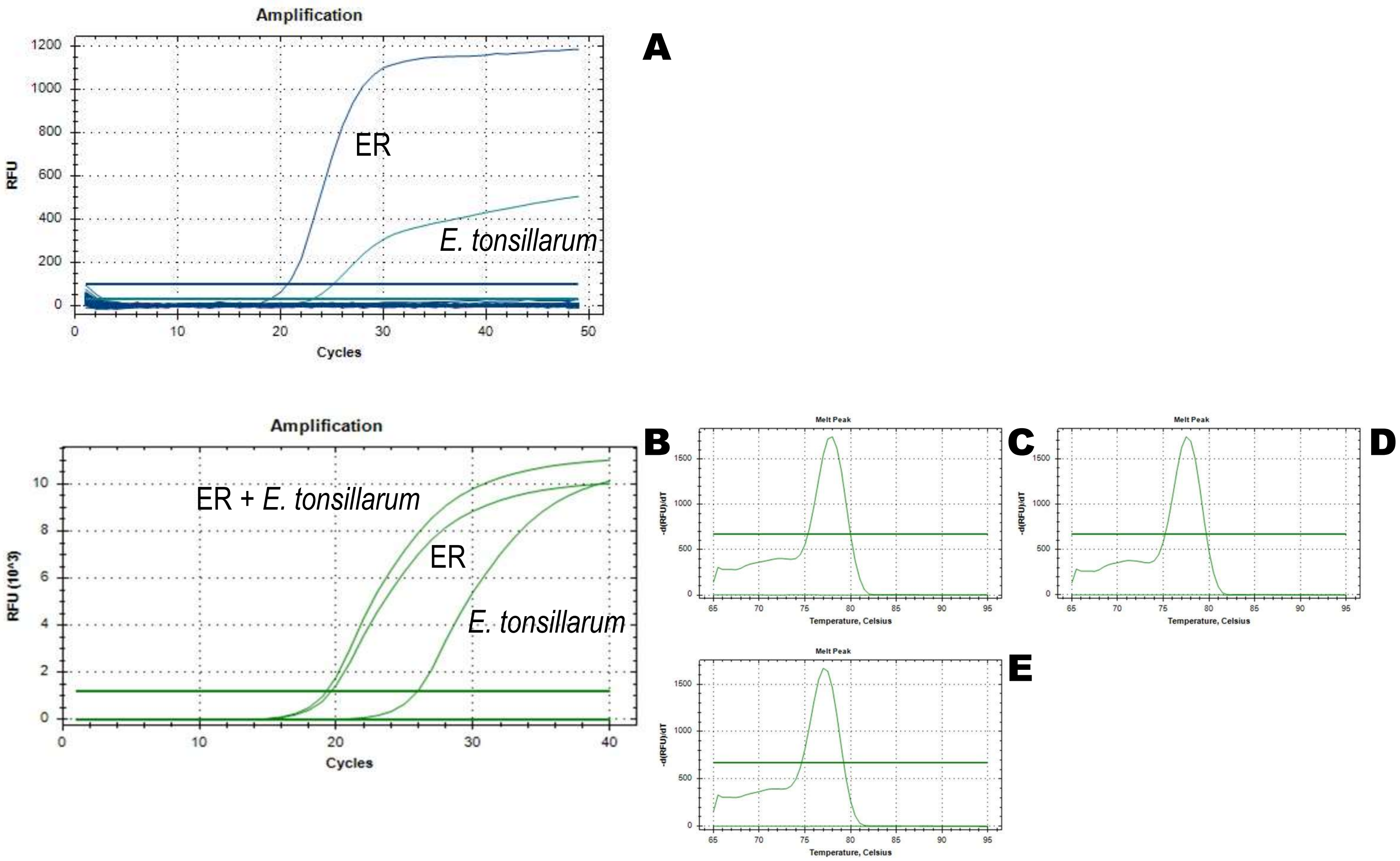

Primer and probe sequences used in probe based real-time PCR (probe) and SYBR-green based real-time PCR (SYBR) assays **F**

Name	Sequence	probe	SYBR
Ery4423F	5′-ATTTCTCTAGCAGGTGATTTGG-3′	X	X
Ery4587R	5′-ACCCTCTAATCGATATGCATCA-3′	X	X
E. rhusiopathiae probe	5′-FAM-AACGAAACGATTAGTAGTCCAACA-BHQ-1′	X	-
E. tonsillarum probe	5′-TET-AAATATTCATGAGACAATCAGCAGT-BHQ-1′	X	-
E. sp. strain 2 probe†	5′-TR-CGAAGGGTTTAAATATTTCTGAGAC-BHQ-2′	X	-

X used in assay, - not used in assay

F, forward; R, reverse; BHQ, Black Hole Quencher; FAM, 6-carboxyfluorescein; TET, tetrachlorofluorescein; TR, Texas Red-X

† Now named *E. piscisicarius.*

Supplementary figure 2. A) Representative example of probe based real-time PCR for ER, *E. tonsillarum* and *E. piscisicarius* with positive controls for ER and *E. tonsillarum* indicated. B) Representative example of SYBR-green based real-time PCR using primers described in F) with positive controls for a mixture of ER and *E. tonsillarum*, ER alone and *E. tonsillarum* alone indicated. Melting curves of positive controls C) a mixture of ER and *E. tonsillarum* (78.0˚C), D) ER alone (77.5˚C) and E) *E. tonsillarum* alone (77.0˚C) in the SYBR-green based real-time PCR shown in B). F) Descriptions of primers and probes used in these assays originally published in reference #32 Pal N, Bender JS, Opriessnig T. Rapid detection and differentiation of *Erysipelothrix* spp. by a novel multiplex real-time PCR assay. Journal of applied microbiology. 2010;108(3):1083-93.

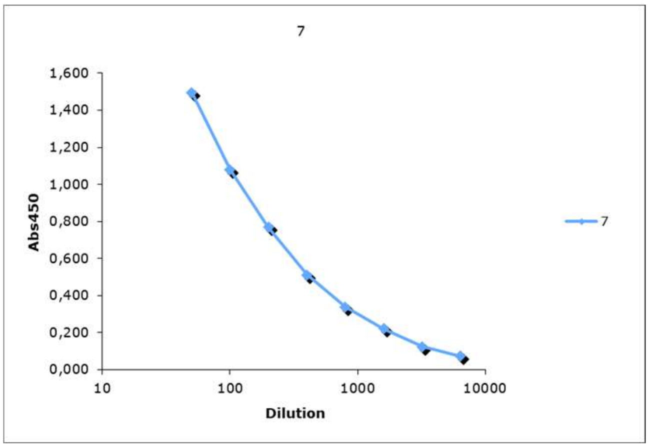

Values for the linear part of the curve, in red values used in regression analysis		
#7		
log10 dilution	dilution	Abs A450-A650
1,698970	50	1,495
2,000000	100	1,079
2,301030	200	0,768

SUMMARY OUTPUT ***from regression analysis***

Regression Statistics	
Multiple R	0,99651885
R Square	0,993049818
Adjusted R Square	0,986099635
Standard Error	0,042988545
Observations	3

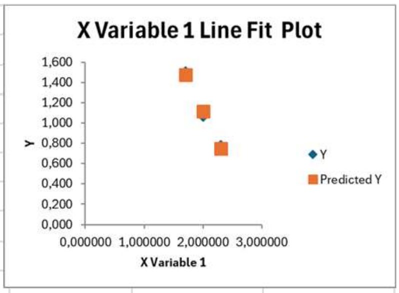

ANOVA

	df	*SS*	*MS*	*F*	*Significance F*
Regression	1	0,264046445	0,264046	142,8811	0,053135232
Residual	1	0,001848015	0,001848		
Total	2	0,26589446			

	Coefficients	*Standard Error*	*t Stat*	*P-value*	*Lower 95%*	*Upper 95%*	*Lower 95,0%*	*Upper 95,0%*
**Intercept**	3,528145147	0,203475941	17,33937	0,036675	0,942738186	6,113552107	0,942738186	6,113552107
**X Variable 1**	-1,207022573	0,100978282	-11,9533	0,053135	-2,490073293	0,076028147	-2,490073293	0,076028147

RESIDUAL OUTPUT

Observation	*Predicted Y*	*Residuals*
1	1,47745	0,01755
2	1,1141	-0,0351
3	0,75075	0,01755

Equation to calculate titer (dilution) at Abs=1:

$$10^{((1\text{-Intercept})/\text{X variable 1})}$$

in this example:

$$10^{((1-3.528)/-1.207)} = \mathbf{124}$$

Supplementary figure 3: An example of calculation of IgY titer defined as theoretical dilution to achieve an absorbance$_{450\text{-}650}$ of 1. Shown are results from a serum sample from chicken #7 tested by ELISA with *H. filiformis* antigen (for details see Material and methods sections 8.5 and 8.6). Top left diagram shows the dilution curve for absorbance values and regression analysis was performed on the values of the linear part of the curve (top right). The output of the regression analysis is shown below along with the calculation of titer using the Intercept and X Variable 1 values from the regression analysis.

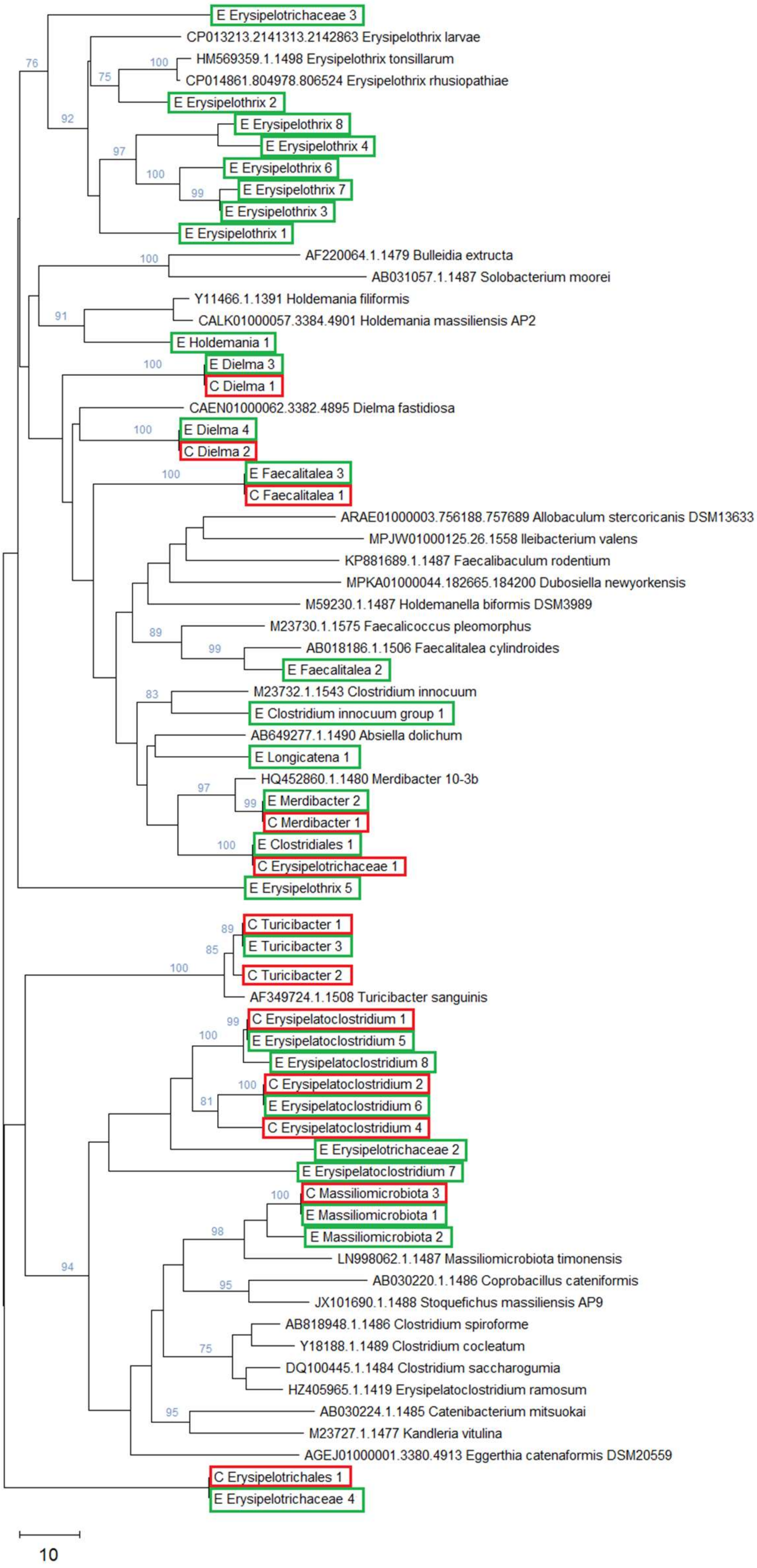

Supplementary Figure 4. Partial 16S rRNA sequences of putative *Erysipelotrichaceae* observed in chicken intestinal samples (red) and environmental samples (green) in the present study with selected reference sequences (no colour highlight), compared with the Neighbor-Joining algorithm. Bootstrap values (in % from 1000 replicates) are shown for branch separations supported by ≥75%.

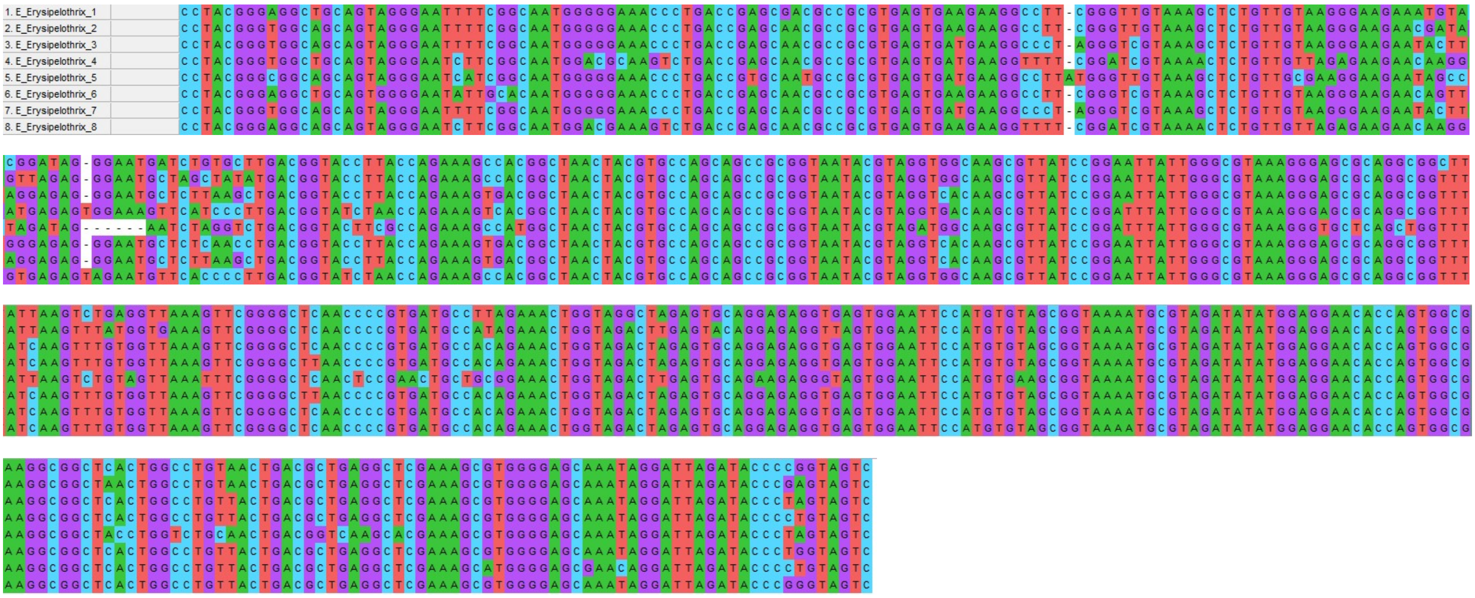

Supplementary figure 5: Multiple alignment of partial 16S rRNA sequences from putative *Erysipelothrix* spp. observed in this study
